# The relationship between physical function and psychological symptoms in Parkinson's disease: A systematic review and meta‐regression analysis

**DOI:** 10.1111/cns.14562

**Published:** 2024-02-09

**Authors:** Philip Hodgson, Alastair Jordan, Charikleia Sinani, Divine Charura, Samuel T. Orange

**Affiliations:** ^1^ Tees, Esk and Wear Valleys NHS Foundation Trust York St John University York UK; ^2^ York St John University York UK; ^3^ Newcastle University Newcastle upon Tyne UK

**Keywords:** meta‐regression, Parkinson's disease, physical, psychological, symptom interaction, systematic review

## Abstract

**Aims:**

This systematic review and meta‐regression aimed to examine available literature reporting measures of physical function, anxiety, and/or depression and whether any relationships exist between these measures in individuals with Parkinson's disease.

**Methods:**

MEDLINE, CINAHL, AMED, and APA PsychInfo databases were systematically searched. Screening, quality assessment, and data extraction were completed alongside meta‐regression analysis.

**Results:**

Of 1175 studies retrieved, 40 were selected for analysis with only one study assessing the relationship between physical and psychological outcomes within their cohort. A total of 27 studies were also eligible for meta‐regression analysis—a total sample of 1211 participants. Meta‐regressions of five combinations of paired physical and psychological outcomes showed a significant moderating effect of symptoms of depression (Beck Depression Inventory) on mobility (Timed‐Up‐and‐Go test; coefficient = 0.37, 95% CI 0.09 to 0.65, *p* = 0.012) and balance (Berg Balance Score) scores (coefficient = −1.25, 95% CI −1.77 to −0.73, *p* < 0.001).

**Conclusion:**

Although physical and psychological outcomes of interest were used in all included studies, only one examined their relationship. Our analysis suggests that symptoms of depression may influence measures of mobility and balance. Specifically, as the severity of symptoms of depression increases, performance on measures of mobility and balance worsens.

## Graphical abstract

Summary of studies collecting data for both physical and psychological outcome measures in Parkinson's disease. Although commonly collected together, it is extremely uncommon for the relationship between physical and psychological symptoms commonly experienced by people with Parkinson's disease to be investigated.

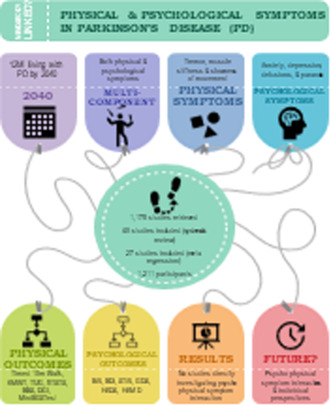

## 1 INTRODUCTION

Parkinson's disease (PD) is a multifaceted neurodegenerative condition impacting upon many aspects of an individual's physical and psychological well‐being. PD is the second most common neurodegenerative disease,^1^ and the most common neurodegenerative movement disorder worldwide,^2^ with prevalence tending to increase with age.^3^ It is expected that by 2040, the global incidence of PD will exceed 12 million.^4^ The three main physical symptoms of PD are tremors, muscle stiffness, and slowness of movement.^5^ In addition, PD can impact an individual's mental well‐being, with symptoms of depression, delusions, paranoia, hallucinations, and PD‐associated dementia commonplace resulting directly from PD itself or through medication side effects.^6^

It is well documented that individuals with PD present with higher incidences of mental health (MH) problems such as depression, anxiety, schizophrenia, and psychotic symptoms when compared to the general population.^7^, ^8^ More specifically, up to 40% of people with PD (PwPD) will have depression^9^ or anxiety,^10^ whereas this figure is only 17% in the general population.^11^ Current NICE guidelines^12^ do not address or provide specific recommendations for MH problems in this population, instead referring to existing generic guidelines on depression in adults with chronic health problems and how to access allied health professionals (e.g., physiotherapists and PD nurse specialists). This is a striking contrast to other neurological conditions such as multiple sclerosis, where guidelines include specific recommendations for regular cognitive, emotional, or MH screening.^13^, ^14^

Evidence in older populations suggests a relationship between physical and psychological presentations.^15^ There is, however, a paucity of evidence to substantiate such a relationship in the PD population. From available evidence, it has been suggested that PwPD feel that anxiety may amplify their physical symptoms,^16^ and when they become more anxious the incidences of freezing of gait increase.^17^ To add to this, a number of studies have suggested that as anxiety increases, so does the severity of motor symptoms as assessed using the Unified Parkinson's Disease Rating Scale (UPDRS).^18^, ^19^, ^20^, ^21^ Although these studies show that there might be an association between physical function and psychological symptoms, it is not well understood. It should also be noted that the UPDRS is a global measure of PD severity and includes both motor and non‐motor domains. This relationship is yet to be confirmed in more specific measures of physical function such as balance and mobility, or considering other psychological symptoms associated with PD.^22^, ^23^ It is our belief that there is likely to be an intrinsic link between physical and psychological symptoms in PD, with the purpose of this review being to investigate this based on work completed to date.

A narrative review published in 2018 suggested the need for further research to better understand the influence of non‐motor symptoms on gait and function in PD.^24^ To our knowledge, there has been no systematic review of literature reporting outcomes for both physical and psychological measures in individuals with PD. Given the proportion of individuals with PD impacted by psychological symptoms, future work has the potential to improve the understanding of any interaction between physical and psychological symptoms.

This systematic review and meta‐regression analysis aimed to examine available literature reporting outcome measures of physical function, anxiety, and depression; and whether any relationships exist between these measures in individuals with PD. Prior to undertaking this review, it was hypothesized that while many studies commonly collect data for both physical and psychological outcomes in PD research, there will be limited evidence exploring the potential relationship between these outcomes.

## 2 METHODS

This review was reported following the preferred reporting items for systematic reviews and meta‐analyses (PRISMA) guidelines^25^ and the review protocol was registered on PROSPERO: CRD42021281392. Ethical approval was obtained from York St John University (STHEC0045).

### 2.1 Search strategy

Database searching was undertaken on 10/23/2021. PH independently and systematically searched four databases (MEDLINE, CINAHL, AMED, and APA PsychInfo) from their date of inception until 10/22/2021. A consistent search strategy was used for each database. Table 1 provides details of the search terms used. Reference lists of all included articles were screened to identify additional studies of interest.

**TABLE 1:** Search terms used and level searched.

1. Keyword (Abstract)		2. Keyword (Abstract)		3. Keyword (All Text)		4. Keyword (All Text)
Parkinson*	AND	“Mental Health”	AND	Physiotherap*	AND	“Activit* of Daily Living”
OR		OR		OR		OR
PD		“Mental Illness*”		Rehab*		ADL
		OR		OR		OR
		“Mental Disorder”		Therap*		“Everyday Tasks”
		OR		OR		OR
		“Psych* Illness”		“Physical Activit*”		“Routine Tasks”
		OR		OR		OR
		Anxi*		Exercis*		Functio*
		OR				
		Depression				
		OR				
		“Psychiatric Disorder”				
		OR				
		SMI				
**5. Keyword (All Text)**		**6. Keyword (Abstract)**				
Well‐being	NOT	“Personality Disorder”				
OR						
Wellbeing						
OR						
“Quality of Life”						
OR						
QOL						
OR						
“Standard of Living”						
OR						
Independ?nce						

### 2.2 Inclusion and exclusion criteria

Following database searching, remaining articles were screened as summarized by the PICOS acronym. **P**articipants: Diagnosis of PD, aged 18 or older. **I**ntervention: Not applicable. **C**omparison: Not applicable. **O**utcomes: Inclusion of a physical and psychological outcome measure of interest (see Table 2). **S**tudy design: All quantitative study designs are included. Only papers published in peer‐reviewed journals and written in English were included, with language screening initially undertaken using automation tools available within the database and subsequently checked manually.

**TABLE 2:** Outcome measures.

**Physical outcome measures**	
Gait	10 MW (10 m Walk Test), 6MWT (6 Minute Walk Distance), Rapid Turns, M‐PAS (Modified Parkinson Activity Scale), and TUG (Timed‐Up‐And‐Go Test)
Balance	Push and Release, Berg Balance Scale, M‐PAS Chair (Modified Parkinson Activity Scale–Chair), FTSTS (Five Times Sit to Stand), M‐PAS Gait (Modified Parkinson Activity Scale–Gait), TUG (Timed‐Up‐And‐Go Test), Rapid Turns, DGI (Dynamic Gait Index), FGA (Functional Gait Assessment), and Mini‐BESTest (Mini Balance Evaluation Systems Test)
Transfers	M‐PAS Bed (Modified Parkinson Activity Scale–Bed), M‐PAS Chair (Modified Parkinson Activity Scale–Chair), FTSTS (Five times Sit to Stand), and TUG (Timed Up‐And‐Go Test)
Physical Capacity	6MWT (6 Minute Walk Distance) with Borg 6–20
**Psychological outcome measures**	
Anxiety	PAS (Parkinson's Anxiety Scale), BAI (Beck Anxiety Inventory), STA (State‐Trait Anxiety Inventory), GAD‐7 (Generalized Anxiety Disorder Assessment), and HADS (Hospital Anxiety and Depression Scale)
Depression	Depression–BDI‐II (Beck Depression Inventory‐II), Ham‐D (Hamilton Depression Rating Scale), PHQ‐9 (Patient Health Questionnaire), GDS‐15 (Geriatric Depression Scale), and HADS (Hospital Anxiety and Depression Scale)

The physical outcome measures of interest were based on the recommendations by the European Physiotherapy Guideline for Parkinson's disease,^26^ whereas the psychological outcomes were selected from those used in clinical settings alongside pilot searching of previous work in the area. Physical outcomes include: 10‐Minute Walk Test (10 MW)^27^; 6‐Minute Walk Test Distance (6MWT)^28^; Rapid Turns^29^; Modified Parkinson Activity Scale (M‐PAS)^30^; Timed‐Up‐And‐Go Test (TUG)^31^; Push and Release^32^; Berg Balance Scale (BBS)^33^; Five times Sit to Stand (FTSTS)^34^; Dynamic Gait Index (DGI)^35^; Functional Gait Assessment (FGA)^36^; and Mini‐Balance Evaluation Systems Test (Mini‐BEST).^37^ Psychological outcomes include: Anxiety—Parkinson's Anxiety Scale (PAS)^38^; Beck Anxiety Inventory (BAI)^39^; State‐Trait Anxiety Inventory (STAI)^40^; Generalized Anxiety Disorder Assessment (GAD‐7)^41^; Hospital Anxiety and Depression Scale (HADS)^42^; Depression—Beck Depression Inventory‐II (BDI‐II)^43^; Hamilton Depression Rating Scale (HAM‐D)^44^; Geriatric Depression Scale (GDS‐15)^45^; and Hospital Anxiety and Depression Scale (HADS).^42^ All outcome measures were selected based on existing evidence demonstrating good validity and reliability within the PD population.

Two researchers (PH and AJ) independently screened the titles and abstracts of relevant literature before completing further full‐text screening to assess eligibility. Both screening stages were completed using an Excel workbook customized for the review process. Any disagreement was discussed, and if a consensus could not be reached, guidance was available from a third reviewer (CS). Studies were grouped by outcome and then by outcome pairings for synthesis. All relevant studies identified were included in the systematic review; however, only incidences where five or more studies used the same combination of physical and psychological outcomes were included in the meta‐regression analysis. This decision was based on evidence that the 95% confidence interval (CI) included the final estimate in 83% of meta‐analyses after five studies.^46^

### 2.3 Data extraction and quality assessment

Data extraction was performed by two reviewers (PH and AJ) independently. Discrepancies were resolved through discussion, with a third reviewer (CS) available for consultation if necessary. A standardized pre‐piloted Excel spreadsheet was used to extract the following data: lead author; publication date; country; population; study design; intervention type; sample size; age; stage of PD; and details of physical and psychological outcome measures used, including group‐level mean and SDs at baseline. It was necessary to extract group‐level data as many studies did not report whole‐sample mean and SD data. Baseline data for each outcome of interest was used to avoid the impact of any study interventions. Authors were contacted in cases of missing or unclear information where required.

The Quality Assessment Tool for Quantitative Studies developed by the Effective Public Health Practice Project (EPHPP) was used to assess the quality of included studies.^47^ This tool evaluates seven domains: selection bias; study design; confounders; blinding; data collection method; withdrawals and drop‐outs; and intervention integrity. Each domain is scored as strong, moderate, or weak, and studies can be classified as strong, moderate, and weak overall. This tool has appropriate content and construct validity as well as good intra‐ and inter‐rater reliability.^48^, ^49^

Initial training, independent scoring, and consensus discussion for two papers were completed by all assessors prior to full scoring being undertaken. Concurrent and independent quality assessment was performed by two reviewers (PH and AJ). Any disagreements between reviewers were resolved through discussion, with a third reviewer available (CS) when necessary.

### 2.4 Analysis

Study authors were contacted to request raw data or correlation coefficients for the physical and psychological outcomes of interest; however, a lack of responses precluded a meta‐analysis of correlation coefficients. Consequently, meta‐regression analyses of group‐level data for physical and psychological outcomes were completed to test whether the psychological outcome score at baseline moderated the physical function score at baseline.^50^ Physical function data from each group (mean and variance) were pooled in a random‐effects, multi‐level meta‐analysis that accounted for non‐independence of data. The model was fitted with the maximum‐likelihood estimation and studies were weighted according to the inverse of the sampling variance. The meta‐analysis produced a weighted average in the physical outcome score across all studies. The mean psychological outcome scores in each group were then entered into the meta‐analysis model as a covariate, which allowed us to test whether the psychological scores statistically moderated the physical function weighted average. Analyses were completed for the following variables used in the same studies: 6MWT and BDI; BBS and BDI; TUG and BDI; TUG and GDS; and BBS and GDS. The *p*‐value from Cohen's Q‐test was used as a measure of heterogeneity. Statistical analyses were conducted in R version 4.0.02 (R Foundation for Statistical Computing). Statistical significance was set at *p* < 0.05. Data are presented from the meta‐regression as model coefficient with corresponding 95% CI and *p*‐value.

## 3 RESULTS

### 3.1 Literature selection

**FIGURE 1:**
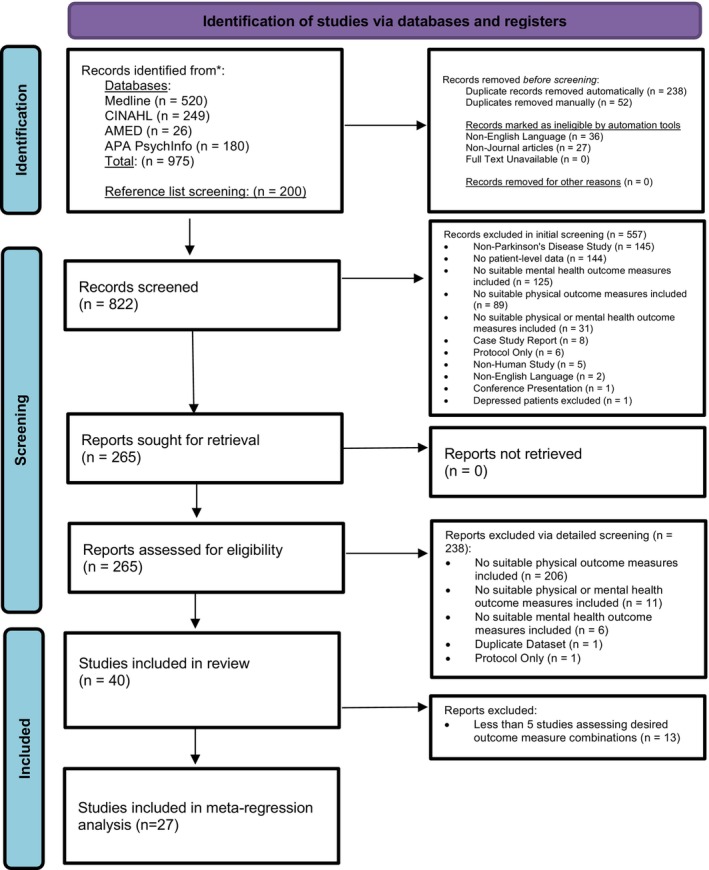
PRISMA flow diagram.

Figure 1 shows the PRISMA flow diagram. In total, 1175 studies were identified, with 265 progressing to full‐text review. The systematic review consisted of 40 studies,^51^, ^52^, ^53^, ^54^, ^55^, ^56^, ^57^, ^58^, ^59^, ^60^, ^61^, ^62^, ^63^, ^64^, ^65^, ^66^, ^67^, ^68^, ^69^, ^70^, ^71^, ^72^, ^73^, ^74^, ^75^, ^76^, ^77^, ^78^, ^79^, ^80^, ^81^, ^82^, ^83^, ^84^, ^85^, ^86^, ^87^, ^88^, ^89^, ^90^ consisting of 18 randomized controlled trials,^52^, ^54^, ^57^, ^58^, ^60^, ^61^, ^66^, ^67^, ^68^, ^69^, ^70^, ^71^, ^76^, ^78^, ^79^, ^81^, ^83^, ^87^ 5 clinical control trials,^53^, ^63^, ^65^, ^72^, ^89^ 11 cohort studies,^55^, ^56^, ^59^, ^62^, ^73^, ^75^, ^82^, ^84^, ^85^, ^86^, ^90^ 2 cohort analytic studies,^74^, ^77^ 1 case–control study,^51^ and 3 other.^64^, ^80^, ^88^ Twenty‐seven of these studies^51^, ^52^, ^53^, ^54^, ^55^, ^56^, ^57^, ^58^, ^59^, ^60^, ^61^, ^62^, ^63^, ^64^, ^65^, ^66^, ^67^, ^68^, ^69^, ^70^, ^71^, ^72^, ^73^, ^74^, ^75^, ^76^, ^77^ were eligible for inclusion in the meta‐regression analysis giving a total sample of 1211 PD participants.

### 3.2 Study and participant characteristics

Key study details including title, authors, and year published are summarized in Table 3. This table also includes details of the study population, exclusion criteria, and outcome measures completed within the study.

**TABLE 3:** Study characteristics.

Article Title	Author(s)	Year published	Intervention setting	*n*	Population	Exclusion criteria	Primary outcome	Relevant physical outcome measures used	Relevant mental health outcome measures used	Baseline physical outcomes			Baseline psychological outcomes	
										6MWT	TUG	BBS	BDI	GDS
Physical activity in patients with Parkinson's disease: a holistic approach based on the ICF model	Aktar B, Balci B, Donmez Colakoglu B	2020	No intervention	60 (25 sedentary PD, 35 non‐sedentary PD)	PD H&Y Stages <4	Standardized Mini‐Mental State Examination score < 24; H&Y > 4, be unable to walk with help or without help for at least 10 min, neurological condition (other than PD), orthopedic and cardiovascular disease; surgical history related to PD	ICF Domains	6MWT, TUG	BDI, BAI	Sedentary Group: Median = 397.20 m (IQR = 319.95–456.60, Mean = 387.02 +/− 85.16); Non‐Sedentary Group: Median = 440.10 m (IQR = 393.60–490.50, Mean = 445.78 +/−70.83)	Sedentary Group: Median = 8.86 m (IQR = 7.63–10.88, Mean = 9.62 +/−2.76); Non‐Sedentary Group: Median = 7.65 m (IQR = 6.93–8.29, Mean = 7.78 +/−1.2)		Sedentary Group: Median = 11.00 (IQR = 5.50–16.50, Mean = 12.32 +/− 9.41); Non‐Sedentary Group: Median = 11.00 (IQR = 7.00–16.00, Mean = 11.31 +/− 6.66)	
A randomized controlled cross‐over trial of aerobic training versus Qigong in advanced Parkinson's disease	Burini D, Farabollini B, Iacucci S, Rimatori C, Riccardi G, Capecci M, Provinciali L, Ceravolo MG	2006	Small groups (Location unclear)	26 PD patients	PD H&Y Stages 2–3	Severe cognitive impairment (MMSE < 24), concomitant severe neuro, cardio, or orthopedic disorder, contraindications for CPET or aerobic training, physio, or rehab 2 months prior	UPDRS	6MWT	BDI, PDQ‐39	Group 1: 419 m +/− 65 m (mean +/− SD); Group 2: 405 m +/− 44 m			Group 1: 10 +/− 0–29 (median +/− Range); Group 2: 15 +/− 0–29	
The efficacy of exercise programs for Parkinson's disease: Tai Chi versus combined exercise	Cheon SM, Chae BK, Sung HR, Lee GC, Kim JW	2013	Group (Location unclear)	23 Female PD patients	PD H&Y Stages 2–3	Severe motor complications, dementia, or psychiatric symptoms that would prevent participating in exercise program regularly	Unclear	6MWT	BDI	Combined Exercise: 362.1 m +/− 62.1 m; Tai Chi: 342.8 m +/− 63.9 m; Control: 354.8 m +/−47.6 m			Combined Exercise: 27.6 +/− 12.3; Tai Chi: 23.4 +/− 10.2; Control: 23.2 +/− 18.1	
Multicenter, randomized controlled trial of PDSAFE, a physiotherapist‐delivered fall prevention program for people with Parkinson's disease	Chivers Seymour K, Pickering R, Rochester L, Roberts HC, Ballinger C, Hulbert S, Kunkel D, Marian IR, Fitton C, McIntosh E, Goodwin VA, Nieuwboer A, Lamb SE, Ashburn A	2019	Home based	474 PD patients	PD H&Y Stages 1–4	Unclear	Risk of repeating falls	Mini‐BESTest, 5TSTS	GDS, PDQ‐39					
Effects of a Nordic Walking program on motor and non‐motor symptoms, functional performance, and body composition in patients with Parkinson's disease	Cugusi L, Solla P, Serpe R, Carzedda T, Piras L, Oggianu M, Gabba S, Di Blasio Andrea, Bergamin M, Cannas A, Marrosu F, Mercuro G	2015	City Park	20 Idiopathic PD patients	PD H&Y Stages 1–3	MMSE < 24, unavailability for the intervention, debilitating or vision impairment impeding full participation, and disorders affecting clinical assessment of disease	Unclear	6MWT, 5TSTS, BBS, TUG	BDI	NW Group: 330.9 m +/−62.9 m (mean +/− SD); Control Group: 328.1 m +/− 59.8 m	NW Group: 8.8 +/− 2.8 (mean +/‐SD); Control Group: 9.2 +/− 2.3	NW Group: 44.2 +/− 7.8 (mean +/‐SD); Control Group: 45.2 +/− 6.9	NW Group: 14.8 +/− 9.2 (mean +/‐SD); Control Group: 14.4 +/− 11.9	
Effects of an adapted physical activity program on motor and non‐motor functions and quality of life in patients with Parkinson's disease	Cugusi L, Solla P, Zedda F, Loi M, Serpe R, Cannas A, Marrousa F, Mercuro G	2014	Group (Location unclear)	9 Idiopathic PD patients	PD H&Y Stages 1–3	MMSE < 24, unavailability for the intervention, debilitating or vision impairment impeding full participation, and disorders affecting clinical assessment of disease	Unclear	6MWT, 5TSTS, BBS, TUG	BDI	304.1 m +/− 121.7 m (mean +/− SD)	10.8 +/− 3.4 (mean +/− SD)	36.1 +/− 9.5 (mean +/‐SD)	20.8 +/− 9.8 (mean +/‐SD)	
The effects of arm crank training on aerobic capacity, physical performance, quality of life, and health‐related disability in patients with Parkinson's disease	Daƒü Figen, √áimen √B, Doƒüu O	2021	Unclear (likely clinic/gym based)	13 Idiopathic PD patients	PD H&Y Stages 1–3	Receipt of physio/rehab 6 months prior, regular exercise, chronic metabolic, cardiopulmonary, and neuromuscular diseases	Cardiorespiratory and physical performance evaluation	6MWT, TUG	BDI, PDQ‐39	476.76 m +/− 57.06 m (mean +/− SD), 477 (360–560) (median, min–max)	9.94 +/− 2.05 (mean +/− SD), 9.65 (8.05–15.80) (median, min–max)		20.84 +/− 8.69 (mean +/‐SD), 19.00 (8.00–35.00) (median, min‐max)	
Resistance training reduces depressive symptoms in elderly people with Parkinson's disease: a controlled randomized study	de Lima TA, Ferreira‐Moraes R, Alves WMGDC, Alves TGG, Pimentel CP, Sousa EC, Abrahin O, Cortinhas‐Alves EA	2019	Unclear (likely clinic/gym based)	33 Idiopathic PD patients	PD H&Y Stages 1–3	MMSE < 24, unstable cardiovascular disease, neuro, cardio, orthopedic conditions, and uncontrolled diseases that impede safe engagement in exercise	Depressive Symptoms	TUG	HAM‐D, PDQ‐39					
Predictors of physical activity levels in individuals with Parkinson's disease: a cross‐sectional study	Feliciano JS, Rodrigues SMA, de Carvalho Lana R, Polese JC	2021	No intervention	50 Idiopathic PD patients	PD H&Y Stages 0–4	For other neuro diseases, MMSE < 13 if illiterate, 18 if elementary and middle education, or 26 if HE	Unclear	Mini‐BESTest	PHQ‐9					
The effects of functional training, bicycle exercise, and exergaming on walking capacity of elderly patients with Parkinson's disease: a pilot randomized controlled single‐blinded trial	Ferraz DD, Trippo KV, Duarte GP, Neto MG, Bernardes Santos KO, Filho JO	2018	Outpatient clinic	62 Idiopathic PD patients	PD H&Y Stages 2–3	Visual or hearing impairment, joint and muscle disease preventing exercise, chronic uncontrolled disease (hypertension, diabetes, myocardial infarction, angina, arrhythmias, substance abuse, and contraindications for exercise (ACSM)) Exercise prog 6 months prior and resistance training 12 months prior	6MWT	6MWT, 10 m Walk Test	GDS, PDQ‐39					
KICK OUT PD: Feasibility and quality of life in the pilot karate intervention to change kinematic outcomes in Parkinson's disease	Fleisher JE, Sennott BJ, Myrick E, Niemet CJ, Lee M, Sanghvi M, Liu Y, Ouyang B, Hall DA, Comella CL, Chodosh J	2020	Community group	15 PD patients	PD H&Y Stages 1–3	>20 miles from participating in karate studio, atypical PD, H&Y > 4, severe psychiatric disorders, severe anxiety or depression requiring inpatient hospitalization or suicidal ideation within 30 days, previous participation in martial arts or boxing programs, and inability to commit to attending 2× classes for 10× weeks	Feasibility	TUG	HADS					
Effectiveness of a dance–physiotherapy combined intervention in Parkinson's disease: a randomized controlled pilot trial	Frisaldi Elisa, Bottino P, Fabbri M, Trucco M, De Ceglia A, Esposito Nadia, Barbiani D, Camerone EM, Costa F, Destefanis C, Milano E, Massazza G, Zibetti M, Lopiano L, Benedetti F	2021	Group (Location unclear)	38 Idiopathic PD patients	PD H&Y Stages 1–2	H&Y > 2, UPDRS P3 > 32, cognitive impairment, severe orthopedic comorbidities, and used walking aids, Could not guarantee their presence for the whole study period	UPDRS	6MWT, TUG, Mini‐BESTest	BDI, STAI, PDQ‐39	Experimental Group: 497 m +/− 76.69 m (mean +/− SD), 500 (480:551) (median, Q1:Q3); Control Group: 526 m +/− 91.54 m, 541 (475:594) (median, Q1:Q3)	Experimental Group: 7.22 +/− 1.15 (mean +/− SD), 7 (6:8) (median, Q1:Q3); Control Group: 6.56 +/− 1.22, 6 (6:8) (median, Q1:Q3)		Experimental Group: 6.53 +/− 5.06 (mean +/‐SD), 5 (2:9) (median, Q1:Q3); Control Group: 7.84 +/− 7.46, 5 (3:14) (median, Q1:Q3)	
Body awareness training in the treatment of wearing‐off‐related anxiety in patients with Parkinson's disease: results from a pilot randomized controlled trial	Ghielen I, van Wegen EEH, Rutten S, de Goede CJT, Houniet‐de Gier M, Collette EH, Burgers‐Bots IAL, Twisk JWR, Kwakkel G, Vermunt K, van Vliet B, Berendse HW, van den Heuvel OA	2017	Group (Location unclear)	38 PD patients	PD H&Y Stages 2–3	MMSE < 24, insufficient motivation, other neuro, orthopedic, cardiopulmonary disease	Self‐efficacy	10 m Walk Test	BAI, BDI, PDQ‐39					
Nordic Walking and Walking in Parkinson's disease: a randomized single‐blind controlled trial	Granziera S, Alessandri A, Lazzaro A, Zara D, Scarpa A	2021	Group—Hospital Rehab Garden	32 PD patients	PD H&Y Stages 2–3	Parkinsonism (multisystemic atrophy, supranuclear progressive palsy, Parkinson–dementia, vascular parkinsonism), Mini‐Mental Parkinson Test <24/30, any comorbidity contraindicating moderate‐intensity physical exercise, and other neurological diseases with motor involvement	UPDRS	6MWT, 10 m Walk Test, TUG	BDI, PDQ‐39	Total Sample: 415.7 m +/− 109.7 m (mean +/− SD); Treatment Group: 389.4 m +/− 122.3 m; Control Group: 442.0 m +/− 91.7 m	Total Sample: 12.1 +/− 14.9 (mean +/− SD); Treatment Group: 15.9 +/−‐ 20.6; Control Group: 8.4 +/− 2.3		Total Sample: 11.2 +/− 6.9 (mean +/− SD); Treatment Group: 11.3 +/− 5.5; Control Group: 11.1 +/− 8.3	
Functional outcomes of an integrated Parkinson's disease wellbeing program	Horne JT, Soh D, Cordato DJ, Campbell ML, Schwartz RS	2020	Group (Location unclear)	135 PD patients	PD H&Y Stages 1–3	Significant neuropsychiatric disturbance including severe depression, Significant cognitive impairment	Unclear	TUG, 10 m Walk Test, BBS	PDQ‐39, DASS‐21					
Exercise management using a mobile app in patients with Parkinsonism: prospective, open‐label, single‐arm pilot study	Kim A, Yun SJ, Sung K‐S, Kim Y, Jo JY, Cho H, Park K, Oh B‐M, Seo HG	2021	Patient‐determined location (Home‐based)	21 PD and atypical Parkinsonism patients	PD H&Y Stages 2–4	Severe cog or physical impairment, Hoehn 5, meeting recommended exercise	Exercise amount	BBS, TUG	GDS, PDQ‐39		13.87 +/− 7.82 (mean ± SD), range = 7–42	48.38 +/− 9.84 (mean +/‐SD), range = 21–56		9.48 +/− 3.42 (mean +/‐SD)
Effects of group, individual, and home exercise in persons with Parkinson's disease: a randomized clinical trial	King LA, Wilhelm J, Chen Y, Blehm R, Nutt J, Chen Z, Serdar A, Horak FB	2015	Various	58 Idiopathic PD patients	Mild or Severe PD	Need assistance with ADL, unable to speak or read in English, other exercise study 12 months prior, >10 h/week, participate in conflicting study, mod‐severe cog impairment, and lack transportation	PPT	Mini‐BESTest, TUG	GDS, PDQ‐39		Home: 11.3 +/− 0.9 (mean +/− SD); Individual: 10.9 +/−‐ 3.5; Class: 13.2 +/−‐ 8.9			Home: 7.4 +/− 4.8 (mean +/− SD); Individual: 9.5 +/− 4.9; Class: 7.0 +/− 5.9
Turo (Qi Dance) program for Parkinson's disease patients: randomized, assessor blind, waiting‐list control, partial crossover study	Lee HJ, Kim SY, Chae Y, Kim MY, Yin C, Jung WS, Cho KH, Kim SN, Park HJ, Lee H	2018	Unclear, Potentially unsupervised	32 PD patients	PD H&Y Stages 1–3	Other neurological or cognitive impairments (K‐MMES 4 20), having received any exercise therapy within the preceding 3 months	UPDRS	BBS	BDI			Turo Group: 53.0 +/− 2.5 (mean +/− SD); Control Group: 53.2+/−3.3	Turo Group: 11.2 +/− 7.2 (mean +/‐SD); Control Group: 13.3 +/− 7.7	
A structural model of health‐related quality of life in Parkinson's disease patients	Lee J, Choi M, Jung D, Sohn YH, Hong J	2015	No intervention	217 PD patients	Not advanced PD	Cognitive impairment, dementia, and bedbound	UPDRS	BBS (Korean)	GDS			50.88 +/− 5.86 (mean +/− SD), Range = 15–56		5.72 +/− 4.33 (mean +/‐SD), Range = 0–15
Impacts of an exercise program and motivational telephone counseling on health‐related quality of life in people with Parkinson's disease	Lee J, Choi M, Yoo Y, Ahn S, Jeon JY, Kim JY, Byun JY	2020	Group (Sports facility) plus telephone	42 PD patients	PD H&Y Stages 0–3	Medication change within 4 weeks, cog impairment, severe neuro, orthopedic, cardiopulmonary, mental disorders, and exercise 2× week for 30 min or more.	Unclear	TUG, BBS, 6MWT	GDS		Experimental Group: 9.44 +/− 1.52 (mean +/− SD); Control Group: 9.41 +/− 2.83	Experimental Group: 52.32 +/− 2.77 (mean +/− SD); Control Group: 51.89 +/− 3.40		Experimental Group: 7.45 +/− 5.28 (mean +/‐SD); Control Group: 3.45 +/− 3.53
Neural networks associated with quality of life in patients with Parkinson's disease	Nakano T, Kajiyama Y, Revankar GS, Hashimoto R, Watanabe Y, Kishima H, Ikeda M, Mihara M, Mochizuki H, Hattori N	2021	No intervention	247 PD patients	PD ‐ Stage(s) unclear	Unclear	Unclear	TUG, BBS, 10 m Walk Test	HAM‐D, GDS, PDQ‐39		Unavailable	Unavailable		Unavailable
Health‐related quality of life and physical function in individuals with Parkinson's disease after a multidisciplinary rehabilitation regimen—a prospective cohort feasibility study	Nielsen C, Siersma V, Ghaziani E, Beyer N, Magnusson SP, Couppé C	2020	Inpatient rehabilitation	214 PD patients	PD H&Y Stages 1–3	Psychiatric or geriatric, requiring day care, medicine or drug addiction, prior rehabilitation offered previously, other neuro diseases	PDQ‐39	TUG	HADS					
Effects of robotic treadmill training on functional mobility, walking capacity, motor symptoms, and quality of life in ambulatory patients with Parkinson's disease: a preliminary prospective longitudinal study	Paker N, Bugdayci D, Goksenoglu G, Sen A, Kesiktas N	2013	Outpatient clinic	10 Idiopathic PD patients	PD H&Y Stages 1–3	Cognitive or cooperation disorder, another neurological disorder, uncontrolled hypertension, orthostatic hypotension, cardiovascular system disorder, or rigidity that interferes with walking or deep brain stimulator implantation	Functional mobility and walking capacity	TUG, 10 m Walk Test	HADS, PDQ‐39					
DRUM‐PD: the use of a drum circle to improve the symptoms and signs of Parkinson's disease (PD)	Pantelyat A, Syres C, Reichwein S, Willis A	2016	Community group	20 PD patients	PD ‐ Stage(s) unclear	Unable to consent, did not have objective bradykinesia on UPDRS, unable to walk or stand without support, unable/willing to participate for full 6 weeks	Quality of Life	TUG	PDQ‐39, GDS		Drum Group: 10.2 +/− 2.6 (mean +/− SD); Control Group: 7.8 +/− 1.1			Drum Group: 3.0 +/− 4.5 (mean +/‐SD); Control Group: 0.4 +/− 0.7
Nordic walking and free walking improve the quality of life, cognitive function, and depressive symptoms in individuals with Parkinson's disease: a randomized clinical trial	Passos‐Monteiro E, B Schuch F, T Franzoni L, R Carvalho A, A Gomeñuka N, Becker M, Rieder CRM, Andrade A, G Martinez F, S Pagnussat A, A Peyré‐Tartaruga L	2020	University setting	33 pd patients	PD H&Y Stages 1–4	Surgical procedures within 6 months, heart disease or uncontrolled BP, myocardial infarction within 12 months, pace maker, stroke, neuro disease, acute pain, or prosthesis making walking impossible.	Quality of Life	BBS	GDS			NW Group: 51.19 +/− 1.2 (mean +/‐SD); FW Group: 47.44 +/− 2.5		NW Group: 2.8 +/− 0.3 (mean +/‐SE); FW Group: 4.6 +/− 0.4 (mean +/‐SE)
Tango for treatment of motor and non‐motor manifestations in Parkinson's disease: a randomized control study	Rios Romenets S, Anang J, Fereshtehnejad S‐M, Pelletier A, Postuma R	2015	Dance studio	40 Idiopathic PD patients	PD H&Y Stages 1–3	Patients unable to stand for at least 30 min or walk for ≥3 m without an assistive device, dementia (defined according to MDS dementia criteria), severe hearing and vision problems, change in dopaminergic therapy over the preceding 3 months, serious medical conditions which precluded dancing or could be worsened by exercise, more than 3 falls in the 12 preceding months, and other medical conditions which could affect study participation (e.g., drug abuse/alcoholism)	UPDRS	TUG, Mini‐BESTest	BDI		Tango Group: 7.4 +/− 2.0 (mean +/‐SD); Control Group: 7.9 +/− 2.5		Tango Group: 7.9 +/− 6.6 (mean +/‐SD); Control Group: 7.7 +/− 5.3	
Exercise increases caudate dopamine release and ventral striatal activation in Parkinson's disease	Sacheli MA, Neva JL, Lakhani B, Murray DK, Vafai N, Shahinfard E, English C, McCormick S, Dinelle K, Neilson N, McKenzie J, Schulzer M, McKenzie DC, Appel‐Cresswell S, McKeown MJ, Boyd LA, Sossi V, Stoessl AJ	2019	Group (Location unclear)	35 PD patients	PD H&Y Stages 1–3	In Supplemental Online Material	fMRI	TUG	BDI		Aerobic Group: 9.94 +/− 1.52 (mean +/‐SD); Control Group: 11.95 +/− 5.12		Aerobic Group: 8.21 +/− 6.26 (mean +/‐SD); Control Group: 9.77 +/− 8.06	
Sardinian folk dance for individuals with Parkinson's disease: a randomized controlled pilot trial	Solla P, Cugusi L, Bertoli M, Cereatti A, Della Croce U, Pani D, Fadda L, Cannas A, Marrosu F, Defazio G, Mercuro G	2019	Group (Location unclear)	20 PD patients	PD H&Y Stages 0–3	H&Y > 3, dementia, atypical Parkinson's disease, drugs not for PD, complementary disability or autonomic issues, health contraindications	UPDRS	6MWT, BBS, 5TSTS, TUG	BDI	Dance Group: 330.7 m +/− 120.48 m (mean +/− SD); Control Group: 333.28 m +/− 120.07 m	Dance Group: 6.9 +/− 1.04 (mean +/‐SD); Control Group: 7.43 +/− 1.18	Dance Group: 40.0 +/− 3.5 (mean +/‐SD); Control Group: 37.3 +/− 5.2	Dance Group: 14.10 +/− 3.45 (mean +/‐SD); Control Group: 13.67 +/− 4.47	
The effects of mindfulness meditation‐based complex exercise program on motor and nonmotor symptoms and quality of life in patients with Parkinson's disease	Son HG, Choi E‐O	2018	Community group	63 PD patients	PD H&Y Stages 1–3	>3 H&Y Scale, Clinically unstable, Unable to communicate, unable to walk independently, previous experience of alternative therapies	Unclear	6MWT	GDS, STAI					
Self‐reported depression and anxiety are correlated with functional disability in Parkinson's disease	Still A, Hale L, Swain N, Jayakaran P	2021	No intervention	19 PD patients	PD H&Y Stages 1–4	Comorbidities affecting gait and balance, unable to follow instructions, >4 on 1.1 of MDS‐UPDRS	Unclear	DGI	HADS					
Vastly different exercise programs similarly improve Parkinsonian symptoms: a randomized clinical trial	Tollár J, Nagy F, Hortobágyi T	2019	Small groups–Hospital outpatient physio gym	74 PD patients	PD H&Y Stages 2–3	MMSE < 24, BDI > 40, severe cardiac disease, uncontrolled diabetes, stroke, brain injury, seizure disorder, deep brain stimulation, ortho surgery, pacemaker, hemophilia, motor fluctuations, and LD dyskinesia.	UPDRS	BBS, BESTest, DGI, 6MWT	BDI	Exergaming: 204.6 m +/− 34.94 m (mean +/− SD); Cycling: 222.4 m +/− 40.85 m; Control: 270.2 +/− 90.66		Exergaming: 23.6 +/− 3.60 (mean +/− SD); Cycling: 22.7 +/− 4.24; Control: 26.3 +/− 5.21	Exergaming: 12.4 +/− 2.75 (mean +/− SD); Cycling: 12.7 +/− 3.24; Control: 12.4 +/− 2.94	
A high‐intensity multicomponent agility intervention improves Parkinson patients' clinical and motor symptoms	Tollár J, Nagy F, Kovács N, Hortobágyi T	2018	Small groups–Hospital gym	55 PD patients	PD H&Y Stages 2–3	Cognitive impairment (Mini‐Mental State Examination score < 24), depression (Beck Depression Inventory score > 40), severe cardiac disease (including congestive heart failure, ischemic disease, presence of pacemaker, and orthostatic hypotension), uncontrolled diabetes, history of stroke, traumatic brain injury, seizure disorder, or current participation in a self‐directed or formal group exercise program	MDS‐UPDRS (M‐EDL)	TUG	BDI, PDQ‐39		Experimental Group: 16.1 +/− 3.7 (mean +/‐SD); Control Group: 18.6 +/− 4.2		Experimental Group: 17.0 +/− 5.3 (mean +/‐SD); Control Group: 18.0 +/− 10.6	
A pilot study to evaluate multi‐dimensional effects of dance for people with Parkinson's disease	Ventura MI, Barnes DE, Ross JM, Lanni KE, Sigvardt KA, Disbrow EA	2016	Group (Location unclear)	15 PD patients	PD H&Y Stages 1–2	History of stroke, significant head trauma, prior neurosurgery, significant vision impairment, atypical PD, and MMSE < 25	Feasibility/acceptability	TUG	GDS		Intervention Group: 11.8 +/− 2.0 (mean +/‐SD); Control Group: 17.9 +/− 8.0			Intervention Group: 4.4 +/− 2.4 (mean +/‐SD); Control Group: 6.3 +/− 2.6
Dance for PD: a preliminary investigation of effects on motor function and quality of life among persons with Parkinson's disease (PD)	Westheimer O, McRae C, Henchcliffe C, Fesharaki A, Glazman S, Ene H, Bodis‐Wollner I	2015	Group–Dance centre based	12 Idiopathic PD patients	PD H&Y Stages 1–4	Unclear	UPDRS	BBS	BDI, PDQ‐39			Better Functioning Group: 54.5 +/− 1.9 (mean +/‐SD); Worse Functioning Group: 41.0 +/− 12.6	Better Functioning Group: 5.7 +/− 4.4 (mean +/‐SD); Worse Functioning Group: 15.3 +/− 11.6	
Effect of simplified Tai Chi exercise on relieving symptoms of patients with mild‐to‐moderate Parkinson's disease	Zhu M, Zhang Y, Pan J, Fu C, Wang Y	2019	Inpatient/Outpatient (location unclear)	41 Idiopathic PD patients	PD H&Y Stages 1–3	Neuro deficit other than PD, severe dementia, inability to understand protocol, psychiatric illness, unable to walk unaided, medication‐affecting balance or attention, current participation in exercise prog	UPDRS	BBS	HAM‐D, PDQ‐39					
Short‐term effectiveness of intensive multidisciplinary rehabilitation for people with Parkinson's disease and their carers	Trend P, Kaye J, Gage H, Owen C, Wade D	2002	Day Hospital	118 PD patients	PD H&Y Stages 1–4	Unclear	Unclear	10 m Walk Test	HADS					
Habitual exercisers versus sedentary subjects with Parkinson's disease: multimodal PET and fMRI study	Sacheli MA, Murray DK, Vafai N, Cherkasova MV, Dinelle K, Shahinfard E, Neilson N, McKenzie J, Schulzer M, Appel‐Cresswell S, McKeown MJ, Sossi V, Jon Stoessl A	2018	No intervention	17 PD patients	PD H&Y Stages 1–3	Atypical PD, cog impairment, depression, unstable cardiovascular/resp disease, osteoarthritis, other neuro disease	Dopamine Release via PET Scan	TUG	BDI		Habitual Exercise Group: 7.55 +/− 0.85 (mean +/‐SD); Sedentary Group: 11.34 +/− 4.09		Habitual Exercise Group: 4.00 +/− 3.94 (mean +/‐SD); Sedentary Group: 11.06 +/− 6.92	
Wii Fit balance board playing improves balance and gait in Parkinson's disease	Mhatre PV, Vilares I, Stibb SM, Albert MV, Pickering L, Marciniak CM, Kording K, Toledo S	2013	Group (Location unclear)	10 Idiopathic PD patients	PD H&Y Stages 2.5–3	MMSE < 24, PD medication change, uncontrolled orthostasis, coronary artery disease, fracture within 6 months, other neuro disease, untreated depression, acute illness, alcohol abuse, sig visual impairment, drug or inherited PD, physio within 1 month, use of Wii fit at home, camptocormia, and contraindications for exercise	Balance	DGI, BBS	GDS			48.8 ± 3.2 (Mean +/− SE)		5.4 ± 1.7 (Mean +/− SE)
Effect of virtual reality dance exercise on the balance, activities of daily living, and depressive disorder status of Parkinson's disease patients	Lee NY, Lee DK, Song HS	2015	Unclear	20 PD patients	PD ‐ Stage(s) unclear	Unclear	Unclear	BBS	BDI			Experimental Group: 46.0 +/− 1.3 (mean +/−SD); Control Group: 45.0 +/− 1.3	Experimental Group: 20.4 +/− 0.9 (mean +/−SD); Control Group: 21.2 +/− 1.3	
Gait velocity and step length at baseline predict outcome of Nordic walking training in patients with Parkinson's disease	Herfurth M Godau J, Kattner B, Rombach S, Grau S, Maetzler W, Berg D	2015	Group (Outdoors)	22 PD patients	PD H&Y Stages 2–2.5	History of concurrent conditions affecting NW training, musculoskeletal, psychiatric problems, and dementia	UPDRS	BBS	BDI			54 (44–56), Median (Range)	1 (1–24), Median (Range)	

Year of publication ranged from 2002^90^ to 2021,^56^, ^57^, ^58^, ^59^, ^64^, ^80^, ^88^ and sample sizes from 9^55^ to 474.^78^ Where reported, the mean group age of participants ranged from 58.23 years^56^ to 72.38 years.^59^ All studies only included PwPD, with only two studies^51^, ^74^ allocating to sedentary versus non‐sedentary groups based on participant self‐reported characteristics.

While 13 studies reported that participants experienced idiopathic PD,^54^, ^55^, ^56^, ^57^, ^60^, ^67^, ^73^, ^75^, ^79^, ^80^, ^81^, ^86^, ^89^ the majority did not report the type of PD. All but five studies^38^, ^40^, ^42^, ^43^, ^54^ reported the Hoehn and Yahr status of participants, with most including participants at stages I to III, and only seven studies^59^, ^66^, ^73^, ^78^, ^80^, ^88^, ^90^ including participants at stage IV.

Exclusion criteria varied across studies. Most common reasons for exclusion included: ongoing cognitive issues such as MMSE score < 24 or dementia; presence of other neurological, cardiovascular, or orthopedic condition; use of mobility aids; frequent falls; inability to read or speak English; or BDI score > 40.

### 3.3 Outcome measures

The most common outcome measures used by included studies were as follows. Physical: 6MWT (13/40 (32.5%)^51^, ^52^, ^53^, ^54^, ^55^, ^56^, ^57^, ^58^, ^63^, ^69^, ^70^, ^81^, ^87^); TUG (22/40 (55.0%)^51^, ^54^, ^55^, ^56^, ^57^, ^58^, ^59^, ^60^, ^63^, ^64^, ^65^, ^67^, ^68^, ^69^, ^71^, ^72^, ^74^, ^79^, ^82^, ^84^, ^85^, ^86^); and BBS (16/40 (40.0%)^54^, ^55^, ^59^, ^61^, ^62^, ^63^, ^64^, ^66^, ^69^, ^70^, ^73^, ^75^, ^76^, ^77^, ^84^, ^85^, ^89^); and Psychological: BDI (19/40 (47.5%)^51^, ^52^, ^53^, ^54^, ^55^, ^56^, ^57^, ^58^, ^61^, ^67^, ^68^, ^69^, ^70^, ^71^, ^73^, ^74^, ^76^, ^77^, ^83^) and GDS (12/40 (30.0%)^59^, ^60^, ^62^, ^63^, ^64^, ^65^, ^66^, ^72^, ^75^, ^78^, ^81^, ^83^, ^87^).

Table 4 shows details of the physical and psychological outcome measures used together within the included studies. The most common physical outcome measures used in conjunction were the 6MWT and BDI (10/40 (25.0%)^51^, ^52^, ^53^, ^54^, ^55^, ^56^, ^57^, ^58^, ^69^, ^70^), TUG and BDI (11/40 (27.5%)^51^, ^54^, ^55^, ^56^, ^57^, ^58^, ^67^, ^68^, ^69^, ^71^, ^74^), TUG and GDS (6/40 (15.0%)^59^, ^60^, ^63^, ^64^, ^65^, ^72^), BBS and BDI (8/40 (20.0%)^54^, ^55^, ^61^, ^69^, ^70^, ^73^, ^76^, ^77^), and BBS and GDS (6/40 (15.0%)^59^, ^62^, ^63^, ^64^, ^66^, ^75^).

**TABLE 4:** Combination of physical and psychological outcome measures reported.

		Psychological outcome measures							
		PAS	BAI	STAI	GAD‐7	HADS	BDI	HAM‐D	GDS
Physical outcome measures	Timed 10 m Walk	0	1	0	0	2	2	1	2
	6MWT	0	1	2	0	0	10	0	3
	Rapid turns	0	0	0	0	0	0	0	0
	M‐PAS	0	0	0	0	0	0	0	0
	TUG	0	1	1	0	3	11	2	6
	Push and Release	0	0	0	0	0	0	0	0
	BBS	0	0	0	0	0	8	2	6
	FTSTS	0	0	0	0	0	3	0	1
	DGI	0	0	0	0	1	1	0	1
	FGA	0	0	0	0	0	0	0	0
	Mini‐Best	0	0	1	0	0	2	0	2

^a^ This table shows all outcome measure combinations identified through article screening. Only combinations used by 5 or more studies are included in the meta‐regression.

While all studies included in this review collected data for both physical and psychological outcomes, only one directly investigated the potential relationship between physical (DGI) and psychological (HADS) outcomes collected.^88^

### 3.4 Study quality assessment

Initial agreement between reviewers across all seven domains for all studies was 246/280 (87.9%) instances, Kappa = 0.749, indicating substantial inter‐rater reliability.^91^ This level of agreement is in line with previous work^49^ comparing the Cochrane Collaboration Risk of Bias Tool (CCRBT) to the EPHPP.

Sixteen of the 40 studies included in the systematic review were rated as strong (40%^55^, ^57^, ^58^, ^59^, ^60^, ^61^, ^66^, ^69^, ^70^, ^78^, ^81^, ^83^, ^84^, ^85^, ^87^, ^90^), 15 as moderate (37.5%^51^, ^52^, ^53^, ^54^, ^56^, ^62^, ^65^, ^67^, ^71^, ^75^, ^79^, ^80^, ^82^, ^86^, ^89^), and nine as weak (22.5%^63^, ^64^, ^68^, ^72^, ^73^, ^74^, ^76^, ^77^, ^88^). Poor quality was due to recruitment (i.e., unrepresentative sample) *n* = 11,^52^, ^56^, ^63^, ^65^, ^68^, ^73^, ^74^, ^76^, ^79^, ^86^, ^88^ incomplete blinding of assessors *n* = 7,^51^, ^53^, ^63^, ^67^, ^71^, ^72^, ^82^ and difficulty ascertaining withdrawal rates *n* = 4.^54^, ^68^, ^76^, ^77^ For further details, please see Table 5.

**TABLE 5:** Quality assessment.

Article Title	Author(s)	Year Published	A–Selection Bias	B–Study Design	C–Confounders	D–Blinding	E–Data Collection method	F–Withdrawals and Dropouts	G–Intervention Integrity	H–Analysis	Global rating
Physical activity in patients with Parkinson's disease: a holistic approach based on the ICF model	Aktar B, Balci B, Donmez Colakoglu B	2020									Moderate
A randomized controlled cross‐over trial of aerobic training versus Qigong in advanced Parkinson's disease	Burini D, Farabollini B, Iacucci S, Rimatori C, Riccardi G, Capecci M, Provinciali L, Ceravolo MG	2006									Moderate
The efficacy of exercise programs for Parkinson's disease: Tai Chi versus combined exercise	Cheon SM, Chae BK, Sung HR, Lee GC, Kim JW	2013									Moderate
Multicenter, randomized controlled trial of PDSAFE, a physiotherapist‐delivered fall prevention program for people with Parkinson's	Chivers Seymour K, Pickering R, Rochester L, Roberts HC, Ballinger C, Hulbert S, Kunkel D, Marian IR, Fitton C, McIntosh E, Goodwin VA, Nieuwboer A, Lamb SE, Ashburn A	2019									Strong
Effects of a Nordic Walking program on motor and non‐motor symptoms, functional performance and body composition in patients with Parkinson's disease	Cugusi Lucia, Solla Paolo, Serpe R, Carzedda T, Piras Luisa, Oggianu M, Gabba S, Di Blasio A, Bergamin M, Cannas A, Marrosu F, Mercuro G	2015									Moderate
Effects of an adapted physical activity program on motor and non‐motor functions and quality of life in patients with Parkinson's disease	Cugusi L, Solla P, Zedda F, Loi M, Serpe R, Cannas A, Marrousa F, Mercuro G	2014									Strong
The effects of arm crank training on aerobic capacity, physical performance, quality of life, and health‐related disability in patients with Parkinson's disease	Dağ F, Çimen ÖB, Doğu O	2021									Moderate
Resistance training reduces depressive symptoms in elderly people with Parkinson's disease: a controlled randomized study	de Lima TA, Ferreira‐Moraes R, Alves WMGDC, Alves TGG, Pimentel CP, Sousa EC, Abrahin O, Cortinhas‐Alves EA	2019									Moderate
Predictors of physical activity levels in individuals with Parkinson's disease: a cross‐sectional study.	Feliciano JS, Rodrigues SMA, de Carvalho Lana R, Polese, JC	2021									Moderate
The effects of functional training, bicycle exercise, and exergaming on walking capacity of elderly patients with Parkinson's disease: a pilot randomized controlled single‐blinded trial	Ferraz DD, Trippo KV, Duarte GP, Neto MG, Bernardes Santos KO, Filho JO	2018									Strong
KICK OUT PD: feasibility and quality of life in the pilot karate intervention to change kinematic outcomes in Parkinson's disease	Fleisher JE, Sennott BJ, Myrick E, Niemet CJ, Lee M, Sanghvi M, Liu Y, Ouyang B, Hall DA, Comella CL, Chodosh J	2020									Moderate
Effectiveness of a dance–physiotherapy combined intervention in Parkinson's disease: a randomized controlled pilot trial	Frisaldi Elisa, Bottino P, Fabbri M, Trucco M, De Ceglia A, Esposito N, Barbiani D, Camerone EM, Costa F, Destefanis C, Milano E, Massazza G, Zibetti M, Lopiano L, Benedetti F	2021									Strong
Body awareness training in the treatment of wearing‐off related anxiety in patients with Parkinson's disease: results from a pilot randomized controlled trial	Ghielen I, van Wegen EEH, Rutten S, de Goede CJT, Houniet‐de Gier M, Collette EH, Burgers‐Bots IAL, Twisk JWR, Kwakkel G, Vermunt K, van Vliet B, Berendse HW, van den Heuvel OA	2017									Strong
Nordic Walking and walking in Parkinson's disease: a randomized single‐blind controlled trial	Granziera S, Alessandri A, Lazzaro A, Zara D, Scarpa A	2021									Strong
Functional outcomes of an integrated Parkinson's disease well‐being program	Horne JT, Soh D, Cordato DJ, Campbell ML, Schwartz RS	2020									Strong
Exercise management using a mobile app in patients with Parkinsonism: prospective, open‐label, single‐arm pilot study	Kim A, Yun SJ, Sung K‐S, Kim Y, Jo JY, Cho H, Park K, Oh B‐M, Seo HG	2021									Strong
Effects of group, individual, and home exercise in persons with Parkinson's disease: a randomized clinical trial	King LA, Wilhelm J, Chen Y, Blehm R, Nutt J, Chen Z, Serdar A, Horak FB	2015									Strong
Turo (Qi Dance) program for Parkinson's disease patients: randomized, assessor blind, waiting‐list control, and partial crossover study	Lee HJ, Kim SY, Chae Y, Kim MY, Yin C, Jung WS, Cho KH, Kim SN, Park HJ, Lee H	2018									Strong
A structural model of health‐related quality of life in Parkinson's disease patients	Lee J, Choi M, Jung D, Sohn YH, Hong J	2015									Moderate
Impacts of an exercise program and motivational telephone counseling on health‐related quality of life in people with Parkinson's disease	Lee J, Choi M, Yoo Y, Ahn S, Jeon JY, Kim JY, Byun JY	2020									Weak
Neural networks associated with quality of life in patients with Parkinson's disease	Nakano T, Kajiyama Y, Revankar GS, Hashimoto R, Watanabe Y, Kishima H, Ikeda M, Mihara M, Mochizuki H, Hattori N	2021									Weak
Health‐related quality of life and physical function in individuals with Parkinson's disease after a multidisciplinary rehabilitation regimen—a prospective cohort feasibility study	Nielsen C, Siersma V, Ghaziani E, Beyer N, Magnusson SP, Couppé C	2020									Strong
Effects of robotic treadmill training on functional mobility, walking capacity, motor symptoms, and quality of life in ambulatory patients with Parkinson's disease: a preliminary prospective longitudinal study	Paker N, Bugdayci D, Goksenoglu G, Sen A, Kesiktas N	2013									Moderate
DRUM‐PD: the use of a drum circle to improve the symptoms and signs of Parkinson's disease (PD)	Pantelyat A, Syres C, Reichwein S, Willis A	2016									Moderate
Nordic walking and free walking improve the quality of life, cognitive function, and depressive symptoms in individuals with Parkinson's disease: a randomized clinical trial	Passos‐Monteiro E, B Schuch F, T Franzoni L, R Carvalho A, A Gomeñuka N, Becker M, Rieder CRM, Andrade A, G Martinez F, S Pagnussat A, A Peyré‐Tartaruga L	2020									Strong
Tango for treatment of motor and non‐motor manifestations in Parkinson's disease: a randomized control study	Rios Romenets S, Anang J, Fereshtehnejad S‐M, Pelletier A, Postuma R	2015									Moderate
Exercise increases caudate dopamine release and ventral striatal activation in Parkinson's disease	Sacheli MA, Neva JL, Lakhani B, Murray, Danielle K, Vafai N, Shahinfard E, English C, McCormick S, Dinelle K, Neilson N, McKenzie Jessamyn, Schulzer Michael, McKenzie DC, Appel‐Cresswell S, McKeown MJ, Boyd LA, Sossi V, Stoessl AJ	2019									Weak
Sardinian folk dance for individuals with Parkinson's disease: a randomized controlled pilot trial	Solla P, Cugusi L, Bertoli M, Cereatti A, Della Croce U, Pani D, Fadda L, Cannas A, Marrosu F, Defazio G, Mercuro G	2019									Strong
The effects of mindfulness meditation‐based complex exercise program on motor and nonmotor symptoms and quality of life in patients with Parkinson's disease	Son HG, Choi E‐O	2018									Strong
Self‐reported depression and anxiety are correlated with functional disability in Parkinson's disease	Still A, Hale L, Swain N, Jayakaran P	2021									Weak
Vastly different exercise programs similarly improve Parkinsonian symptoms: a randomized clinical trial	Tollár J, Nagy F, Hortobágyi T	2019									Strong
A high‐intensity multicomponent agility intervention improves Parkinson patients' clinical and motor symptoms	Tollár J, Nagy F, Kovács N, Hortobágyi T	2018									Moderate
A pilot study to evaluate multidimensional effects of dance for people with Parkinson's disease	Ventura MI, Barnes DE, Ross JM, Lanni KE, Sigvardt KA, Disbrow EA	2016									Weak
Dance for PD: a preliminary investigation of effects on motor function and quality of life among persons with Parkinson's disease (PD)	Westheimer O, McRae C, Henchcliffe C, Fesharaki A, Glazman S, Ene H, Bodis‐Wollner I	2015									Weak
Effect of simplified Tai Chi exercise on relieving symptoms of patients with mild‐to‐moderate Parkinson's disease	Zhu M, Zhang Y, Pan J, Fu C, Wang Y	2019									Moderate
Short‐term effectiveness of intensive multidisciplinary rehabilitation for people with Parkinson's disease and their carers	Trend P, Kaye J, Gage H, Owen C, Wade D	2002									Strong
Habitual exercisers versus sedentary subjects with Parkinson's disease: multimodal PET and fMRI study	Sacheli MA, Murray DK, Vafai N, Cherkasova MV, Dinelle K, Shahinfard E, Neilson N, McKenzie J, Schulzer M, Appel‐Cresswell S, McKeown MJ, Sossi V, Jon Stoessl A	2018									Weak
Wii Fit balance board playing improves balance and gait in Parkinson's disease	Mhatre PV, Vilares I, Stibb SM, Albert MV, Pickering L, Marciniak CM, Kording K, Toledo S	2013									Moderate
Effect of virtual reality dance exercise on the balance, activities of daily living, and depressive disorder status of Parkinson's disease patients	Lee NY, Lee DK, Song HS	2015									Weak
Gait velocity and step length at baseline predict outcome of Nordic walking training in patients with Parkinson's disease	Herfurth M, Godau J, Kattner B, Rombach S, Grau S, Maetzler W, Berg D	2015									Weak

*Note*: Key: Green = Strong; Orange = Moderate; Red = Weak. Blue colour used to indicate no scoring related to this column.

### 3.5 Meta‐regression

#### 3.5.1 Sample heterogeneity

Cohen's Q‐test confirmed the heterogeneity of the sample in all outcome combinations. 6MWT and BDI: Q (df = 19) = 17,866.1999, *p* < 0.0001; BBS and BDI: Q (df = 13) = 777.0983, *p* < 0.0001; TUG and BDI: Q (df = 19) = 112.3925, *p* < 0.0001; TUG and GDS: Q (df = 9) = 35.5070, *p* < 0.0001; and BBS and GDS: Q (df = 5) = 10.8870, *p* = 0.05.

#### 3.5.2 Coefficients

Analysis showed that higher scores in TUG were associated with higher scores on BDI (coefficient = 0.3675, 95% CI 0.0901 to 0.6450) and GDS (coefficient = 0.4481, 95% CI −0.2111 to 1.1073). Shorter 6MWT distances were associated with higher BDI scores (Coefficient = −2.2732, 95% CI −9.8007 to 5.2543), while lower BBS scores were associated with higher BDI (Coefficient = −1.2518, 95% CI −1.7687 to −0.7349) and GDS scores (Coefficient = −0.2205, 95% CI −1.3136 to 0.8727).

#### 3.5.3 Moderating effects

Analysis showed a significant moderating effect of BDI on BBS (*F* (df1 = 1, df2 = 12) = 27.8439, *p* = 0.0002) and TUG (*F* (df1 = 1, df2 = 18) = 7.7446, *p* = 0.0123). There was no significant moderating effect of BDI on 6MWT. Similarly, there were no significant moderating effects of GDS on TUG or BBS.

**FIGURE 2:**
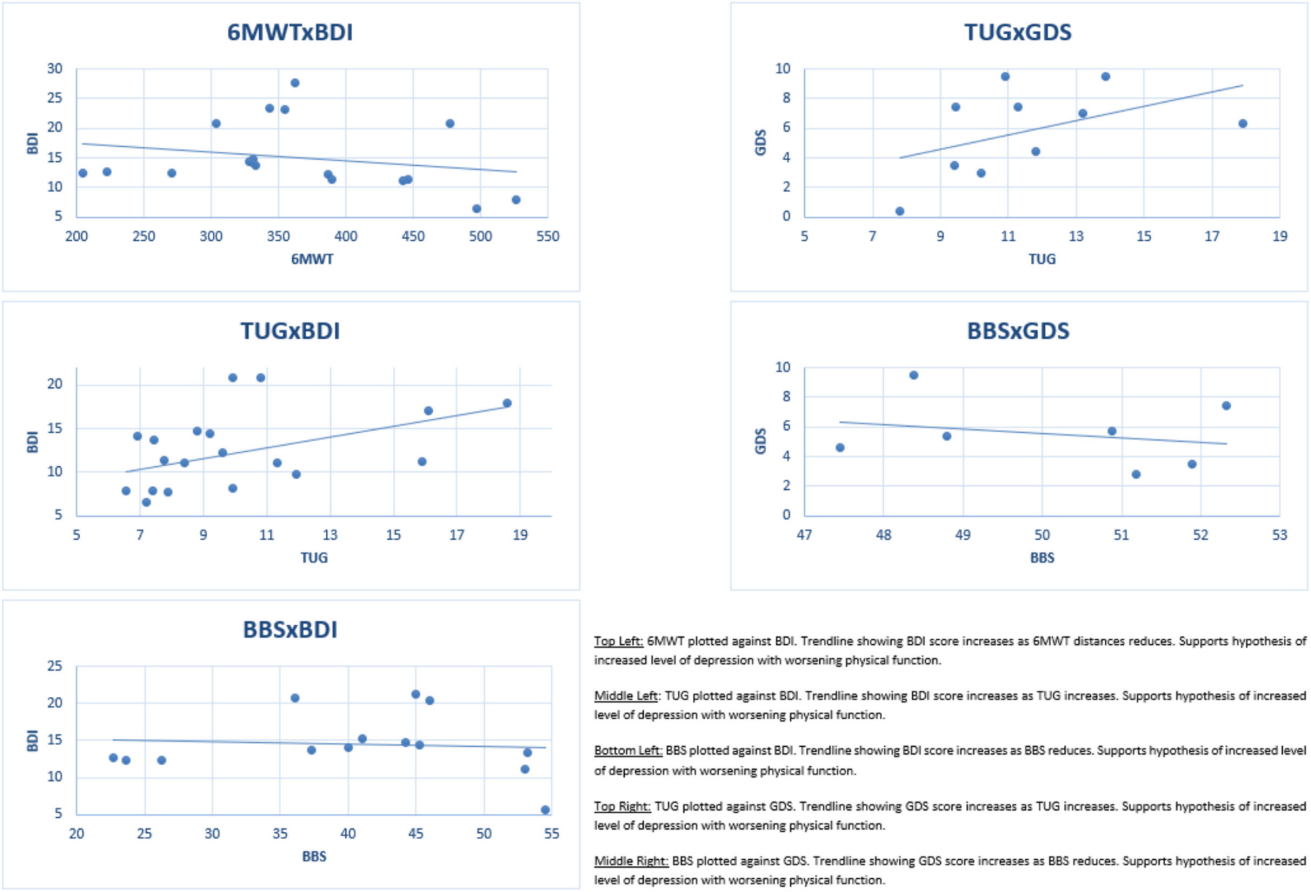
Relationship between physical and psychological outcomes. Top left: 6MWT plotted against BDI. Trendline showing BDI score increases as 6MWT distances reduces. Supports hypothesis of increased level of depression with worsening physical function. Middle left: TUG plotted against BDI. Trendline showing BDI score increases as TUG increases. Supports hypothesis of increased level of depression with worsening physical function. Bottom left: BBS plotted against BDI. Trendline showing BDI score increases as BBS reduces. Supports hypothesis of increased level of depression with worsening physical function. Top right: TUG plotted against GDS. Trendline showing GDS score increases as TUG increases. Supports hypothesis of increased level of depression with worsening physical function. Middle right: BBS plotted against GDS. Trendline showing GDS score increases as BBS reduces. Supports hypothesis of increased level of depression with worsening physical function.

Figure 2 displays extracted group mean data.

## 4 DISCUSSION

To the best of our knowledge, this was the first systematic review and meta‐regression analysis to examine available literature reporting outcome measures of physical function, anxiety, and/or depression and whether any relationships exist between these measures in individuals with PD. Despite many studies routinely collecting data for both physical and psychological outcome measures, only one study examined this relationship.^88^ Our exploratory meta‐regression analysis of extracted baseline group‐level mean data from previous studies suggests a trend for the physical ability of PwPD to reduce as symptoms of depression increase.

Still et al.^88^ completed the only identified study that examined the relationship between physical and psychological outcome measures. Although the authors found significant correlations between participant‐reported physical function and anxiety/depression, this was not the case between clinically assessed physical function and anxiety/depression. More specific, significant correlations were found between self‐reported motor disability (MDS‐UPDRS Part 2) and depression/anxiety (HADS).^88^ Interestingly, this not only supports the hypothesis of a potential interaction between physical and psychological symptoms in PD but also a potential mismatch in this relationship between participant and clinician physical function assessments. Such discrepancy might be due to how individuals with PwPD perceive physical function in comparison to their clinicians.

Our meta‐regression analysis showed that poorer functional ability with gait, balance, and transfers was associated with higher depression scores. Specifically, poorer functional ability with gait and physical capacity (6MWT) were associated with higher depression scores (BDI), while poorer balance (BBS) was associated with higher depression scores (BDI and GDS). Such findings suggest a potential interaction among gait, balance, transfer ability, and physical capacity, with symptoms of depression. The meta‐regression analysis also considered potential moderating effects between outcome measures. Interestingly, a significant moderating effect of depression (BDI) on gait, balance including transfer ability (TUG), and balance (BBS) was found. Our findings suggest that symptoms of depression may directly influence a reduction in physical performance in PwPD evident in outcome measures of gait, balance, and transfer ability. It is possible that such moderating effects may be partially explained by the fact that reduced physical functioning is more likely to increase reliance on others to complete aspects of daily living as the condition progresses.^92^ However, it is important to note that it is difficult to fully interpret such an effect due to the complexity of such interactions. While the meta‐regression analysis conducted as part of this review focuses on the potential influence of psychological distress on physical function, any relationship is likely to be bi‐directional in nature and that limited physical function has the potential to further influence psychological symptoms.

In comparison with Still et al., the results of our meta‐regression analysis suggest that a relationship may exist between clinician‐reported physical and participant‐reported psychological outcome measures. Such contradictory findings may be due to authors using a combination of outcome measures not encountered in other studies and therefore, unable to include within our meta‐regression analysis. To add to this, some of the participants in Still et al.'s study reported psychological symptoms mostly classified as “normal to mild,” and for this reason, their impact on the clinically reported scales might have been minimal.

Non‐motor symptoms including cognitive, mood, autonomic, and sleep disturbances have been observed as a component of PD since its discovery,^93^ and previous work has suggested that even at the earliest stages of PD, non‐motor symptoms may impair patients' functional status and sense of well‐being.^94^ In addition, those who have been living with PD for longer are more likely to experience a larger number of symptoms, with impairment becoming more pronounced as the disease and its symptoms progress over time.^95^ Despite previous research finding significant correlations between anxiety/depression and changes in gait characteristics,^17^, ^96^, ^97^, ^98^, ^99^ it is yet to translate into evidence of anxiety/depression impacting functional or motor disability assessed through clinical outcome measures recommended by the European Physiotherapy Guideline for Parkinson's Disease.^26^

Our systematic review and meta‐regression analysis had several strengths such as the use of multiple reviewers throughout all stages, and the high level of agreement between reviewers due to the comprehensive training prior to appraisal completion. In our review, 77.5% of included studies were rated as either strong or moderate indicating reasonable quality which strengthens our findings. The studies that were rated highly used transparent recruitment methods from multiple sources, fully blinded both participants and assessors to group allocation, and reported withdrawal rates and intervention integrity within their results. This review demonstrates that good‐quality data have been collected as part of previous work, which is a source of untapped potential and would serve as a valuable tool in assessing the extent of any relationship between physical function and psychological symptoms in PD, providing evidence to improve clinical service provision.

While we took steps to strengthen this review, some limitations still remain. The inability to access the raw data or correlation coefficients of the included studies despite requesting these from the corresponding authors meant that meta‐analysis was not possible, and for this reason, meta‐regression analysis of group‐level mean data was our only option. Although the UPDRS^100^ is a commonly used outcome measure, it was not included in the list of outcome measures of interest for two reasons; first, it does not provide the same symptom specificity as the other included outcome measures, and second, it was not listed in the European Physiotherapy Guideline for Parkinson's disease.^26^ We also chose to focus mainly on psychological measures of anxiety and depression excluding others such as apathy, motivation, and fatigue. Several studies excluded participants who had one of the following: cognitive problems; reduced mobility; advanced stage of PD; severe anxiety; and/or depression. Therefore, the findings reported here are based on individuals with less severe physical and psychological problems to preclude us from exploring any psychophysical interactions across the range of PD presentations. Finally, although we used a comprehensive literature search strategy, we did not perform a forward citation searching.

Following this work, it is anticipated that clinicians and researchers will have increased awareness of the potential interaction between physical function and psychological symptoms in PD. A step towards physical and psychological services working more closely together may be for predominantly physical health‐orientated professionals such as PD Nurses, Physiotherapists, and Neurologists/GPs to increase screening for depression in PwPD presenting with lower levels of physical functioning. At this point onward referrals to psychological services could be made in cases where concerns are identified. Improved monitoring of relevant physical outcomes predictive of changes in psychological presentation has the potential to improve the detection and treatment of psychological symptoms experienced by PwPD.

Future research should continue to investigate treatments focused on enhancing physical capacity and improving aspects such as mood. Ideally, researchers will begin to evaluate the relationship between physical and psychological variables within their cohorts, allowing a comprehensive meta‐analysis. In addition, work considering the perspectives of PwPD and clinicians relating to psychophysical symptom interactions would be beneficial. Our final suggestion is for an objective study to monitor changes in both physical function and psychological symptoms over time, alongside investigation of the potential mismatch between how clinician‐rated and participant self‐reported measures of physical function correlate with psychological measures.

## 5 CONCLUSION

This systematic review and meta‐regression analysis highlighted that despite both physical and psychological outcome measure data being routinely collected in many studies in PwPD, only one study included in this review directly examined their relationship. Our exploratory meta‐regression analysis showed a trend for the functional physical ability of PwPD to reduce as scores of depression outcomes increase, showing a significant moderating effect of depression on gait, balance, and transfer performance. This supports the existence of a complex relationship between the physical and psychological symptoms of PD which warrants further investigation, including how any relationship between physical function and psychological symptoms develops as the condition progresses. This work highlights the need for a more integrated mind–body approach to clinical and research practice which can only benefit PwPD. Clinicians with physical health‐focused roles such as PD Nurses, Physiotherapists, and Neurologists/GPs should screen individuals at risk for depression, and refer them to psychological services where needed. Future research is required to uncover the true extent of any psychophysical symptom interaction in PD, alongside considering the views of PwPD about any impact on their daily lives.

## FUNDING INFORMATION

This research was conducted as part of an ongoing PhD project and no specific funding was obtained to support completion of this review.

## CONFLICT OF INTEREST STATEMENT

The authors do not have any financial support or relationships that may pose conflict of interest to disclose.

## PERMISSION TO REPRODUCE MATERIAL FROM OTHER SOURCES

This is an open‐access article under the terms of the Creative Commons Attribution License, which permits use, distribution, and reproduction in any medium, provided the original work is properly cited.

## Data Availability

The data that support the findings of this study are available at 10.25421/yorksj.22600501, 10.25421/yorksj.22600480, and 10.25421/yorksj.22600462. DOIs will become active following publication.
